# Coevolution of a multilayer node-aligned network whose layers represent different social relations

**DOI:** 10.1186/s40649-017-0047-1

**Published:** 2017-11-06

**Authors:** Ashwin Bahulkar, Boleslaw K. Szymanski, Kevin Chan, Omar Lizardo

**Affiliations:** 1 Rensselaer Polytechnic Institute, 110 8th St., Troy, NY 12180 USA; 2 Społeczna Akademia Nauk, Lodz, Poland; 3 US Army Research Laboratory, Adelphi, MD 20783 USA; 4 University of Notre Dame, Notre Dame, IN 46556 USA

**Keywords:** Coevolution of network layers, Machine learning for social network analysis, NetSense dataset

## Abstract

**Background:**

We examine the coevolution of three-layer node-aligned network of university students. The first layer is defined by nominations based on perceived prominence collected from repeated surveys during the first four semesters; the second is a behavioral layer representing actual students’ interactions based on records of mobile calls and text messages; while the third is a behavioral layer representing potential face-to-face interactions suggested by bluetooth collocations.

**Methods:**

We address four interrelated questions. First, we ask whether the formation or dissolution of a link in one of the layers precedes or succeeds the formation or dissolution of the corresponding link in another layer (temporal dependencies). Second, we explore the causes of observed temporal dependencies between the layers. For those temporal dependencies that are confirmed, we measure the predictive capability of such dependencies. Third, we observe the progress towards nominations and the stages that lead to them. Finally, we examine whether the differences in dissolution rates of symmetric (undirected) versus asymmetric (directed) links co-exist in all layers.

**Results:**

We find strong patterns of reciprocal temporal dependencies between the layers. In particular, the creation of an edge in either behavioral layer generally precedes the formation of a corresponding edge in the nomination layer. Conversely, the decay of a link in the nomination layer generally precedes a decline in the intensity of communication and collocation. Finally, nodes connected by asymmetric nomination edges have lower overall communication and collocation volumes and more asymmetric communication flows than the nodes linked by symmetric edges.

**Conclusion:**

We find that creation and dissolution of cognitively salient contacts have temporal dependencies with communication and collocation behavior.

## Background

In this paper we use a dataset containing longitudinal information on a group of individuals in a multilayer node-aligned network to examine dependencies across different types of relations (for details on data collection see [1]). We use this rich source of information, hereafter referred to as the *NetSense data*, to build three distinct network layers linking individuals over time. One is a *nomination* layer constructed from subjective reports of significant contacts, another is a *behavioral* layer constructed from electronic records of communications, and the third is a *behavioral* layer, constructed from bluetooth records indicating spatial collocation of students.

In a previous version of this paper presented at the conference [2], we analyzed dependencies between the first two layers. Here, we aim to understand the relationship between all three layers to shed the light on the link between nominations, communication behavior, and spatial propinquity. This is important, since, as we note in the following section, the exact relationship between these forms of human connectivity is a long-standing, but understudied, problem in social network analysis [3, 4].

## Motivating social science background for the research

Social scientists have traditionally distinguished various dimensions of human connectivity [5–9]. Perhaps the most well-studied dimension of this type in fields like anthropology and sociology is *cognitive saliency*. This can be defined as the subjective prominence of a given contact for an individual at a particular point in time [6, 10, 11]. Empirically, cognitive salience can be measured as the likelihood that an individual will “nominate” another individual as an important contact with regard to a given relation (e.g., friend, advisor, discussion partner, frequent interlocutor). The classical method used to study this dimension of connectivity among individuals is the network survey [6, 7, 12, 13], in which individuals are presented with a “name generator” (to elicit some predetermined number of salient nominees from memory) and a “name interpreter” (to collect relevant information on each nominee). This approach has generated a great deal of knowledge (usually at the level of “ego-networks”), about those contacts who are subjectively the most important to each individual. A key advantage of the NetSense data is the availability of such periodic network surveys recording each participant’s most cognitively salient contacts.

A related dimension of human connectivity is *frequency of interaction* [7, 9, 14]. An important finding of network analysis in sociology is that, while frequency and cognitive salience usually go together [6], the correlation between the two factors is much weaker than would be expected if these two dimensions were two indicators of the same underlying construct such as “tie strength” [7, 9]. Instead, research has established that persons can have high rates of communicate interaction (e.g., established either via observational or self-report methods) with contacts who were not cognitive salient or considered particularly close [5, 7, 8]. In the same way, some non-negligible proportion of the most cognitively salient contacts may be characterized by relatively low rates of communicative frequency [15]. Overall, however, the question of whether frequency of interaction precedes, and therefore leads to, cognitive salience or whether salience leads to more interaction remains a highly debated issue [9, 14–16].

Finally, both classic and more recent work in social networks points to spatial contiguity or *propinquity* as an important indicator of social connectivity [9]. Just like with the relationship between cognitive salience and frequency of interaction, recent work shows that spatial contiguity is an independent dimension of human association, since it can vary independently of the other two factors mentioned. Persons may spent a lot of time (e.g., in workplaces, classrooms, and so on) in spatial proximity to people who are not particularly salient to them or with whom they seldom interact directly [15]. However, research also shows that strong social ties tend to emerge among people who spend time together in the same physical location [16], such that both cognitive salience and frequency of interaction may end up being the result of external factors that channel people into the same physical spaces. This is what [9] has referred to as “proximity” mechanisms governing the formation and maintenance of dyadic connections among individuals.

### Research questions and hypotheses

Social network researchers have proposed studied dependencies between all three of the aforementioned dimensions of human association. Most of this work however, has looked at the connection between two of the aforementioned three dimensions at a time [14, 16], usually with an eye towards establishing the causal precedence of one factor (e.g., frequency of interaction or propinquity) over the other (e.g., the cognitive salience of a given contact). Seldom, however, has the mutual connection between these three factors been explored systematically. We use the rich set of data collected as part of the NetSense project to do just that.

In what follows, we begin by establishing the   validity of the cognitive salience and interaction frequency measures that were collected as part of the NetSense data. We thus begin by exploring the correlations between layers in a multilayer network having three types of links, one based on interaction frequency, another based on collocation frequency, while the third is based on cognitive salience. Social network theory leads us to conjecture that these two dimensions should be positively correlated, with cognitive salient ties displaying (on average) more frequent behavioral interaction than non-salient ties. In essence, we should expect that persons connected in the nomination layer, should have higher interaction frequencies [3, 5, 7, 9].

We then address the question of precedence between interaction frequency and cognitive salience. Drawing on models that see behavior as preceding cognition, we hypothesize that nodes linked by communication edges with large weights at a given point in time should be more likely to appear as cognitively salient contacts in the future. Following [9], we test the same hypothesis with regard to collocation behavior: We thus expect that the more an individual encounters another person in a proximate physical location at a given point in time, the more likely it is that person will appear as a cognitively salient contact in the future. A key issue is which behavioral mechanism, frequency of interaction or collocation, is more important in determining future salience in the nomination [9, 16]. This is an issue that has not been investigated in previous work but that we tackle here using state-of-the-art machine-learning methods.

While the hypothesis that behavior in large part determines cognitive salience is plausible, recent work on the culture-network link also proposes that the reverse arrow of causation is also equally plausible: That going from cognitive salience to behavioral interactions [17, 18]. We propose that a dissolution event in the nomination layer (e.g., being mentioned as a significant contact at time *t* but then not mentioning that contact at time $$t+1)$$ should be a accompanied by decreasing levels of behavioral interaction in terms of both communication and collocation events. Essentially, the disappearance of a nomination link should lead to a gradual decay of intensity of the communication and collocation layers.

We use the temporal features of the NetSense data to examine the question whether *variation* in cognitive salience determines the communication and collocation interactions. We take advantage of the differences in duration among cognitively salient ties (differentiating between newly salient and long-standing ties) hypothesizing that nodes connected by long-standing nomination ties should exhibit higher levels of communication and collocation than the ones linked by newly emerging ties.

In addition, we go beyond establishing differences in behavioral profiles of ties based on their levels of cognitive salience, and examine the question of whether the behavioral temporal signature of social ties differs depending on whether their nodes are linked in the nomination layer or not. We hypothesize that cognitive salient ties will also exhibit behavioral activity in days of the week and times of the day that are associated with informal sociability in this population (e.g., weekends and evenings), and that this feature differentiates them from ties whose nodes are disconnected in the nomination layer.

Finally, we aim to examine, for the first time, coupled asymmetries and non-reciprocities along the nomination and behavioral layers. In traditional networks built from subjective reports of cognitive salience, a common phenomenon is *asymmetry*. This refers to the situation where contact *B* is cognitively salient for person *A*, but not the reverse : *A* is not mentioned by *B* as a cognitive salient contact. Work in social network analysis shows that this situation is fairly common in human social networks, even for ties defined by friendship and intimacy [19]. Here we examine if asymmetries in nominations are connected to non-reciprocities in the two-way flow of communication in the behavioral layer. Recent work also shows that non-reciprocity is more common in human social interactions that would be expected from traditional social and anthropological theory pointing to the “norm” of reciprocity [20]. We hypothesize that these two phenomena are empirically linked: nodes connected by asymmetric nomination edges should be connected by behavioral edges characterized by non-reciprocity. We also connect the phenomenon of cognitive asymmetry with that of behavioral tie decay [21], and hypothesize that asymmetric cognitive ties should exhibit steeper rates of decay in the behavioral communication layer.

## Related work on multilayer network dynamics

A model to generate two social networks synthetically, with both networks coevolving, capturing the properties of both networks is introduced in [22]. A rapidly evolving network based on games is studied in [23]. Nodes in this network have varying incentives to build links. We observe similar behavior in the NetSense data, where certain edges have incentive to develop into an edge in one of the networks, while others do not. A wide swath of previous work, impossible to fully review in this limited space, in social network analysis has investigated dependence and evolution of connections across multiple networks (for a review see [24]).

More generally, multilayer networks have been studied extensively in the Network Science literature. They have been formally defined in [25] which also includes a discussion of related topics such as multilayer networks, multilayer node-aligned networks, multiplex networks, and hypergraphs. The NetSense network can be classified as node-aligned multilayer network because it has one set of nodes and three types of edges connecting these nodes. This reference discusses several properties of multilayer networks and describes how link prediction is done across multilayer heterogeneous networks in [26, 27]. This reference defines also a “meta-path” approach, where sequences of different relations are used as features for link prediction. Novel community detection approaches for evolving multi-slice networks have been discussed in [28] and they will be useful for future work related to our paper.

A study similar in design to that of NetSense is discussed in [29]. The authors analyze a multilayer network of high school students, including a face-to-face interaction layer and a friendship survey layer. They study the difference in structural properties of three layers in their successive paper [30]. Smieszek et al. study a multilayer network between conference participants in [29] including a proximity sensor interaction layer, and the self-reported friendship layer. The authors study in what ways these two layers differ. In contrast to these two papers, our study focuses on the evolution of the edges in our multilayer network. The dataset that contains only records of communication between people is presented in [31]. The participants are high school students transitioning to college. The authors study the change of communication volume between pairs of students as they come into contact with new people, to discover the evolution of communication edges in response to external events. In our study, we focus on how changes of edges in one layer impact edges in other layers. Stopczynski et al. introduce in [32] the Copenhagen dataset defining a multilayer network that includes communication layer, a face-to-face interaction layer, and a Wi-Fi signal-based proximity layer. Several papers on the Copenhagen study describe how different social structures, like groups congregating at the same locations, are discovered using different layers of the network. Large-scale communication record datasets typically focus on basic features, such as call patterns, temporal features of communication records, and evolving communities in such network, as exemplified by [32, 33]. Miritello et al. [34] study how individuals adopt different tie formation strategies to activate and maintain ties. The Copenhagen dataset and the large-scale communication record dataset contain only behavioral edges, while an important element of our study is to investigate how behavioral edges impact changes in the nomination layer.

### Additions to our previous work

The present research builds on, but moves beyond, our previous work, which explored how two-layered social network coevolves in time. This work was published in the *Proceedings of Fifth International Workshop on Complex Networks and their Application* [2]. Here, we introduce a second behavioral network layer, based on the bluetooth collocations of nodes. We demonstrate how the behavioral collocation layer coevolves with the nomination layer (based on nominations) as well as the first communication-based behavioral layer. We then look at the different behavioral stages in the evolution of the networks. In addition, we also included the analysis of temporal dependencies between the two behavioral layers and the nomination layers. Social ties and temporal features have been studied in [35]. However, here we study the temporal dependencies between different kinds of ties and the coevolution of these ties over time.

## NetSense data and the networks

In this section, we introduce the NetSense data  [1] and the networks derived from it. The data were collected at the University of Notre Dame. At the start the Fall semester in 2011, 200 incoming freshmen were enrolled in the NetSense study. Over 150 participated until their graduation in the Spring of 2015. Students participating in the study received free smartphones with unlimited voice and text plans as an incentive for participation. We obtained time-stamped logs of communication records for all study participants. These data contain information on the date, time, and duration (for calls) and character length (for text messages). Data for the first four semesters (lasting from the Fall of 2011 to the Spring of 2013) of the project were available for this study.

In addition to this, we have information about the bluetooth interactions between study participants. The bluetooth interactions are time-stamped. However, the bluetooth interaction data are available only from October 2011 to May 2012. From all the bluetooth interactions, we filter the interactions which are most likely to be face-to-face. Every bluetooth interaction has an associated signal strength value, which is referred to as the received signal strength indicator, or RSSI. The RSSI values vary between 0 and − 120 dB, a value close to 0 implying high quality of the signal. RSSI values above − 65 dB are generally inferred to be most likely face-to-face as mentioned in [36]. Therefore, we consider only the interactions where the RSSI values are above − 65 dB.

Students participating in the NetSense study list up to twenty nominees at the beginning of each semester. Students were asked to list the names of those people with whom they thought they spent the most time communicating or interacting. Recent work has shown that this type of name generator is the most likely to produce an unbiased list of significant contacts [15]. Below, we refer to these cognitively salient contacts as nominees. For each student, nominees could be inside or outside the NetSense study. Because students were asked to also provide the primary phone number of each significant contact, we can link each of those mentioned in the survey to the time-stamped smartphone data.

Accordingly, we propose a model for analyzing the coevolution of multilayer networks whose layers represent different kinds of connections between nodes. We have two behavioral layers, the *communication* layer consists of the edges based on communication records of both telephone calls and text messages between individuals and the *collocation* layer consists of the edges based on bluetooth collocations between individuals. Weights on the edges in the behavioral layers change daily, depending on the volume of communication and the number and length of bluetooth collocations. The *nomination* layer includes edges based on (possibly non-reciprocal) nominations collected via surveys. Edges in the nomination layer may appear and disappear once per semester. Table 1 provides a summary of the three layers of the network.

**Table 1:** A summary of the NetSense layers

Layer	Nodes	Edges	Frequency of evolution
Cognitive salience	Students	Nominations in surveys	Every semester
Collocation behavior	Students	Bluetooth interactions	Evolves continuously
Communication behavior	Students	Calls and texts	Evolves continuously

## Methodology and results

### Dynamic coevolution of the nomination, collocation, and communication layers

In this paper, we are interested in understanding coevolving dynamics between the communication and collocation connectivity layers (indexing behavior) and the layer of nominations (indexing cognitive salience of contacts). We also seek initial validation of these coevolution dynamics. Given that the period of time for which NetSense has gathered information covers the initial stages of a college cohort, we can expect that students will nominate others as significant contacts, communicate, and spend time in proximity of one another within a relatively closed environment. We postulate the existence of multiple relationships between the coevolving collocation, communication, and nomination layers.

We hypothesize that there is a delay between the onset of changing behavior, observed either in communication or collocation, and the nomination of another individual as a cognitively salient contact. Communication (mutual calls or texts) or collocation (i.e., spending time together, whether in a class or in a club or just casual meet-ups) would transition in a later time period to a contact appearing as significant enough to be nominated in the survey. We study how cognitive salience is affected by behavioral interaction whether in terms of communication or collocation. In this section we see how behavior can be used to predict the formation and persistence of cognitive salient relationships. We also expect to see the converse occur, where a decline in cognitive salience leads to a diminishing of communication and collocation.

#### Do nodes connected in the nomination layer have stronger edges in the behavioral layers?

We explore the differences in communication and collocation volumes between nodes who are and who are not connected in the nomination layer. Communication volume is measured by the number of calls and messages exchanged between dyads in a semester, while collocation intensity is measured by the number of bluetooth proximity detection events between the same dyads in a given semester.

**Fig. 1:**
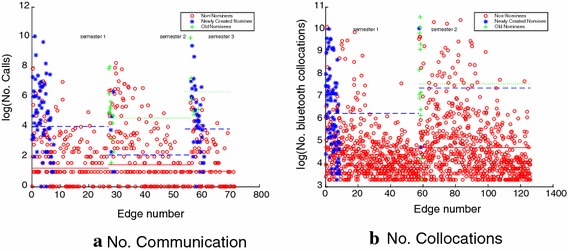
The numbers of communications and collocations for behavioral edges for which the corresponding nomination edge is old (green plus signs), newly created (blue asterisk), and non-existent (red circles). The lines show the average value for the circles of the corresponding color in each semester, the blue dashed line shows the average for newly created edges, the green dotted line shows the average for old edges. The separation is significant between these lines. Generally, edges in which one node nominates the other have corresponding edges with a higher intensity of communication and collocation

As shown in Table 2, we find that nodes linked in the nomination layer (nominees) tend to have behavioral edges with significantly higher volumes of communication and collocations compared to those nodes that do not have a corresponding nomination link, non-nominees). Figure 1a, b illustrates how number of communication events and the number of collocations are distributed among students connected in the nomination layer (blue stars and green crosses) and those who are not mentioned as cognitively salient contacts (red circles). In all, not being mentioned as a cognitively salient contact is associated with lower volumes of communicative interaction and propinquity.

**Table 2:** Difference between nodes connected and disconnected in the nomination layer in terms of weight of their communication and collocation edges

Semester	Nominees			Non-nominees		
	No. calls	No. messages	No. collocations	No. calls	No. messages	No. collocations
Semester 1	70	667	161.2	7	72	36.5
Semester 2	41	915	448.5	12	190	55.6
Semester 3	74	1063	–	5	51	–
Semester 4	34	729	–	4	37	–

#### Do higher weight behavioral edges turn into new nomination edges?

We explore how dynamic changes in the cognitive salience of contacts, as indexed by the formation of new nomination links, correlate with the volume of interaction along the corresponding link in the communication and collocation layer. We examine whether an increase in the cognitive salience of a given contact, is preceded by higher rates of behavioral communication and collocation. We also examine whether links that decline in cognitive salience, as indexed by a dissolution of the corresponding nomination edge, are characterized by lower volumes of communication and collocation the semester before.

**Fig. 2:**
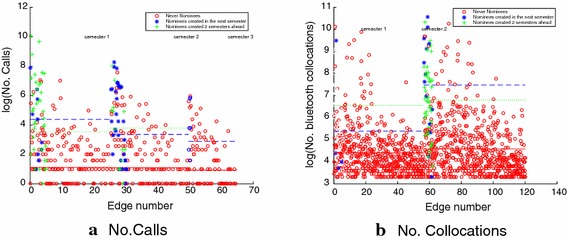
The numbers of calls and collocations between nodes who are to-be-nominees in one semester (blue asterisk), to-be-nominees in two semesters (green plus signs), and not-to-be-nominees (red circles). Generally, to-be-nominees have higher numbers of calls and collocations than not-to-be-nominees. The continuous lines show the average values for the circles of the same color. The separation is large between red and green dotted lines, red and blue lines, but small between dashed blue and dotted green lines. Most of the to-be-nominees edges appear in the first and second semester, since very few new nominations are formed in the fourth semester

We find that contacts that become more cognitively salient by forming a link in the nomination layer are characterized by higher volumes of communication and collocation. Table 3 lists the differences in communication and collocations. Figure 2a, b illustrates how the numbers of calls and collocations are distributed among the to-be-formed and not-to-be-formed nomination edges. In both the cases, we find that communication and collocation volume is higher among the existing and to-be-formed contacts in the nomination layer than among those individuals who do not share a nomination edge.

**Table 3:** Difference in communication volume between pair of nodes who are to-be-nominees and the ones who are not-to-be-nominees

Semester	To-be-nominees			Not-to-be-nominees		
	No. calls	No. messages	No. collocations	No. calls	No. messages	No. collocations
Semester 1	40	407	191.8	5	58	35.7
Semester 2	52	782	195.6	6.5	105	46.7
Semester 3	18	248	–	4	41	–

#### Predicting the formation of links in the nomination layer from the strength of edges in the behavioral layers

Here we examine whether formation and existence of edges in the nomination layer can be predicted from their respective edge weights in the communication and collocation layer. We take the weight of the behavioral edge, defined by the communication or collocation volume, and consider whether behavioral edges above a threshold of the behavior volume can predict whether we observe a formation event in the nomination layer. We expect that edges below the threshold should be more likely not form an edge in the nomination layer in the future.

We measure the performance of this analysis in terms of recall and accuracy. Recall is defined as the ratio of the number of correct prediction to the number of true values in predicted dataset. Accuracy is defined as the ratio of correct predictions to the total number of predictions. We do not use precision as a measure, since the classification is very unbalanced, the number of positive examples is several times smaller than the number of negative examples. Table 4 lists the results for the prediction of existing edges.

**Table 4:** Prediction of nominated contact based on the volume of communication in the communication behavioral layer

Semester	Calls		Messages		Collocations	
	Accuracy (%)	Recall (%)	Accuracy (%)	Recall (%)	Accuracy	Recall
Semester 2	69.3	68.5	73.4	63.1	–	–
Semester 3	73.8	70.5	70.5	81.5	82.1%	76.9%
Semester 4	77.8	75.5	77.9	83.6	–	–

Table 5 lists the results for prediction of future edges. We find that we are able to predict a significant proportion of nomination edges using information from the behavioral layers separately, about 70–80%, with a reasonable accuracy. We are also able to predict a significant proportion of future nomination edges using information from the behavioral layers; about 70–80% of edges are predicted with a reasonable accuracy.

**Table 5:** Prediction of future-nominated contact formation based on volume of communication between the corresponding nodes in the particular semester

Semester	Calls		Messages		Collocations	
	Accuracy (%)	Recall (%)	Accuracy (%)	Recall (%)	Accuracy	Recall
Semester 2	73.1	75.3	74.4	71.3	–	–
Semester 3	72.5	74.4	77.2	78.9	80.2%	75.1%

**Fig. 3:**
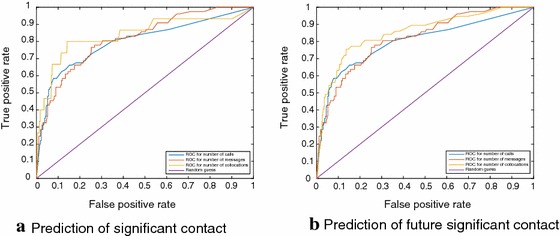
We plot the ROC curves for prediction based on different thresholds of number of calls, messages, and collocations. With a threshold of zero, the false positive rate is 100% and true positive rate (recall) is 100% as well. Moreover, with the threshold equal to the maximum value, the false positive rate is 0% and true positive rate (recall) is 0% as well. We observe that the predictability is significantly higher than random. We see that there is a sharp drop in the false positive rate (which means, increase in accuracy) accompanied by a sharp drop in the recall when the thresholds increase above a certain limit. Behavioral edges where the number of calls, messages, or collocations is above a certain threshold are classified as significant contacts. We observe that as the threshold increases, the false positive rate (FPR) and the true positive rate (TPR) decrease gradually. At a certain higher value of the threshold, the rate of change of both the FPR and TPR increases significantly. This reflects on the distribution of the values of behavioral weights (be it the number of calls, messages, or collocations) of nominees vs. non-nominees and to-be-nominees vs. not-to-be-nominees. Nominees and to-be-nominees are much more likely to have higher behavioral weights, non-nominees and not-to-be-nominees can have higher behavioral weights, but less often. Also, nominees and to-be-nominees are much less likely to have very low behavioral weights

In Fig. 3a, b we show how the performance of prediction changes with changing thresholds. This also reflects upon the differences between the distribution of behavioral weights of nominees and non-nominees and between the to-be-nominees and the not-to-be-nominees.

We then investigate if using both behaviors, communication, and collocation, we can predict the formation of edges in the nomination layer. We use the following features in a machine-learning implementation: number of calls, number of messages, and number of collocations. We predict links in the nomination layer formed in the third semester from the second semester, training the machine-learning model on links formed in the second semester from the first semester. We tried Linear Regression, Support Vector Machines (SVM), SVM–Radial Basis Function (SVM–RBF), and classifiers using Random Forests and k-Nearest Neighbors to predict the evolution of nomination links, but it was Linear Regression that gave us the best results shown in  Table 6 which also demonstrates that using both behavioral data types improves recall compared to using either one alone.

**Table 6:** Difference in recall values when different combinations of behavior are used

Communication only	Collocation only	Collocation + communication
75.3	75.1	81

We also examine whether behavioral edges whose nodes are connected by nomination edges that persist into the next semester tend to have significantly higher weights, as compared to behavioral edges for the nodes with nomination edges that dissolve future. Table 7 lists the differences in communication, while Table 8 shows the differences in the bluetooth collocations. We indeed find that persistent edges in the nomination layer have corresponding communication and collocation edges with significantly weights. The results also show that those nomination edges that are subject to decay tend to have smaller weights in the communication and collocation layers.

**Table 7:** Difference between persistent and dissolving nomination edges in terms of weights of the corresponding communication behavioral edges

Semester	Persistent edges		Dissolving edges	
	No. calls	No. messages	No. calls	No. messages
Semester 1	133.2	1373.1	40.8	492.8
Semester 2	92.1	2137.8	7.3	102.3
Semester 3	75.0	1148.6	8.2	205.9

**Table 8:** Difference between persistent and dissolving nomination edges in terms of the weights of their corresponding collocation behavioral edges

Semester	Persistent edges	Dissolving edges
Semester 1	346.4	116.6
Semester 2	665.8	100.7

#### Do newly formed nomination edges differ from older nomination edges in terms of their communication and collocation volume?

Here we examine whether there are systematic differences between newly formed and older edges in the nomination layer, in terms of how strongly connected they are in the communication and collocation layers. To this end, we measure and compute the difference in the volume of communication and collocation between nodes joined by older (at least one semester old) and newly formed edges in the nomination layer.

As Table 9 shows, we find that nodes connected by older edges in the nomination layer have higher edge weights in the communication layer in comparison nodes connected by edges which have been just been formed in the nomination layer. We also observe that nodes connected by older edges in the nomination layer edges tend to find themselves in the same location more often than nodes connected by the newer edges in the nomination layer edges as shown in Table 10. Note we could calculate collocation results for only one semester due to the limited availability of the bluetooth data.

**Table 9:** Difference in behavioral communication volumes between nodes connected by the old and new nomination edges

Semester	Newly observed nominations		Nomination contact older than one semester	
	No. calls	No. messages	No. calls	No. messages
Semester 2	6	57	61	1340
Semester 3	63	1026	172	2447
Semester 4	7	256	53	1067

**Table 10:** Difference in the numbers of behavioral collocations between nodes connected by the old and new nomination edges

Semester	Newly observed nominations	Nominations older than one semester
Semester 2	67.5	455.6

We also observe that as these newly formed nomination edges age, the nodes connected by them come to have communication volumes similar to, or perhaps slightly higher, than nodes connected by edges in the nomination layer that have existed for a longer time. To shed further light on this issue, we examine communication volumes between nodes connected by the nomination layer in the 3rd and the 4th semesters, and we divide them into edges which were created in the 2nd and the 3rd semesters, respectively, and edges which existed since the 1st semester. We call the former moderately old edges and the latter very old edges. We observe that the nodes connected by the moderately old edges carry on an average of 49 calls and 903 calls, while the nodes connected by the very old edges exchange 29 calls and 795 messages. We infer that communication between nodes that are connected in the nomination edge increases gradually, but then finally stabilizes over a period of time.

#### Is link decay in the nomination layer followed by a weakening of the corresponding edge in the behavioral layers?

In this section we examine the question of whether behavioral links tend to weaken and dissolve after the corresponding edges in the nomination layer decay. To that end, we measure the rate at which pairs of nodes that become disconnected in the nomination layer also become disconnected in the communication and collocation layers. Then we compare that with the rate at which behavioral links dissolve at random. We want to ascertain whether nodes that first experience a dissolution event in the nomination layer are more likely to dissolve edges in the behavioral layers than a random dyad does.

To do this, define the quantity BDND as the average link dissolution rate in the behavioral layers for persons who are not connected in the nomination layer, and BDNC as the average behavioral link dissolution rate for persons that are connected in the nomination layer. In the third and fourth semesters, BDND is significantly greater than BDNC, while the reverse is observed in the second semester. We observe values of 64, 55, and 50% for BDND for the three semesters, and 42, 74, and 62% for BDNC. We also measure the rate at which the nodes connected by edges in the nomination layer that persist into the following semester dissolve their behavioral edges, and denote it as BDNP. We find that BDNP is always 0, meaning that if the nomination link persists then so does the corresponding behavioral link. Yet, we rarely observe dissolution of collocation after the contact is no longer nominated to the list of significant contacts. This implies that people may continue to frequent the same places even after they are no longer cognitively salient contacts.

#### Are the temporal collocation signatures of nodes connected in the nomination layer different from those who are not connected?

In this section we examine the question of whether students who are connected in the nomination layer exhibit distinct patterns of temporal collocation behavior. In particular, we ask whether cognitive salient contacts are more likely to be in the same place during days of the week and times of the day more likely to be associated with sociability (such as weekends and evenings, respectively) than students who do not share a link in the nomination layer. We also look at whether temporal communication and collocation behavior differs for individuals who will become connected in the nomination layer in the future. A positive result here would indicate that we can use observed changes in temporal behavioral signatures among dyads to predict increases in the cognitive salience of a given contact over time.

To do this, we look at different temporal features of the collocation behavior and explore differences among four groups of dyads: nominees (dyads connected in the nomination layer), non-nominees (dyads disconnected in the nomination layer), to-be-nominees (dyads who will form a link in the nomination layer in the future), and not-to-be-nominees (dyads that will in the nomination layer). One key behavioral feature is the number of bluetooth collocations on weekdays versus the number of collocations on weekends. We include Friday evenings into the weekend count. Another is the number of collocations that happen during weekday days versus the number of collocations that happen on weekday evenings and nights. We define collocations that happen in the day, as the ones that happen between 8 a.m. and 6 p.m. and that evening collocations happen after 6 p.m. and before 8 a.m. We observe the behavior differences between nominees and non-nominees and between to-be-nominees and not-to-be-nominees and see if they are similar. Similar behaviors might indicate latent expression of nomination edges.

We observe a significant difference in the temporal patterns of collocation followed by nominees and non-nominees. Non-nominees have interactions largely only on weekdays, while nominees have significant interactions also on weekends. Table 11 lists these differences. On weekdays, non-nominees communicate more in the daytime, while nominees tend to communicate more in the evenings. Table 12 lists these differences.

**Table 11:** Differences between nominees and non-nominees in numbers of collocations between weekdays and weekends

Semester	Nominees		Non-nominees	
	Weekday	Weekend	Weekday	Weekend
Semester 1	97	63	28	8
Semester 2	303	144	42	13

**Table 12:** Differences between nominees and non-nominees in numbers of collocations between evenings and daytimes

Semester	Nominees		Non-nominees	
	Evening	Day	Evening	Day
Semester 1	66	39	12	16
Semester 2	206	108	18	25

We also looked at the differences between to-be-nominees and not-to-be-nominees. We observe a significant difference in the temporal patterns of collocation in the collocation behavioral layer followed by to-be-nominees and not-to-be-nominees. Individuals who remain disconnected in the nomination layer tend to be located in the same place largely on weekdays, while to-be-nominees tend to experience collocation on both weekdays and weekends. Table 13 lists these differences. On weekdays, not-to-be-nominees are more likely to be in the same place in the daytime, while to-be-nominees are more likely to be in the same place in the evenings. In addition, as shown in Table 14, to-be-nominees have temporal collocation behavior patterns that are very similar to current nominees.

**Table 13:** Differences between to-be-nominees and not-to-be-nominees in the numbers of collocations between weekdays and weekends

Semester	To-be-nominees		Not-to-be-nominees	
	Weekday	Weekend	Weekday	Weekend
Semester 1	120	71	27	8
Semester 2	120	76	37	9

**Table 14:** Differences between to-be-nominees and not-to-be-nominees in the numbers of collocations between evenings and daytimes

Semester	To-be-nominees		Not-to-be-nominees	
	Evening	Day	Evening	Day
Semester 1	92	31	11	16
Semester 2	75	55	15	23

### Emergence of cognitively salient contacts through defined stages of interaction

In this section, we examine the dynamic coevolution of the two behavioral layers with respect to the nomination layer. We believe that while both behaviors, communication, and collocation are fairly interchangeable when it comes to prediction of contact formation in the nomination layer, they are not necessarily synchronized and one might precede the other. First, we determine whether collocation is a predictor of future increases in communication, or whether communication predicts increases in future collocation. We then study whether the behavioral factor that follows the other is an intermediary mechanism linking the casually pre-eminent behavioral factor (e.g., collocation or communication) to edge formation in the nomination layer.

#### Emergence of cognitively salient contacts: from collocation to communication to cognitive salience

To look at the evolution of the two behavioral layers, we consider the paths from collocation to significant contact formation occurring through a communication relationship. We would like to know if the communication relationship is an intermediary variable between mere collocation and the formation of link in the nomination layer. We explore whether the formation of a communication edge is correlated with the weight of the corresponding collocation edge. We also examine whether forming or increasing the strength of an collocation edge precedes a corresponding edge creation in the communication layer.

For this purpose, we explore the differences in volume in the collocation layer between nodes that communicate and nodes that do not communicate, and between nodes that are going to become communicators and those that are not going to become communicators. Here, we look at communication edges without the corresponding nomination edges. We can then observe if the number of collocations associated with establishment of a communication relationship is significantly different, perhaps lower, than the number of collocations associated with the establishment of a link in the nomination layer. We also look at pairs of nodes that created communication edges in the second semester, and also became connected in the nomination layer in the third semester. We observe if there has been an increase in the number of collocations from the first to the second semester, and if the number of collocations between them in the second semester differs significantly from the number of collocations between nodes which did not become nominees in the third semester.

**Table 15:** Difference in the numbers of collocations between communicators and non-communicators

Semester	Communicators	Non-communicators
Semester 1	68.9	49.7
Semester 2	116.5	48.8

As shown in Table 15, we observe that dyads that communicate, but are not linked in the nomination layer, tend to experience more bluetooth collocations than dyads that do not communicate with each other. Note that while there is a difference, it is not as drastic as the difference in number of collocations between nominees and non-nominees. As the first column of Table 16 shows, we also find that people who are going to communicate in succeeding semesters, but are not going to be linked in the nomination layer, tend to have more bluetooth collocations than people who will not communicate in the succeeding semesters and also not going to be linked in the nomination layer. Note however, that the number of collocations associated with the establishment of a communication edge is significantly lower than the number of collocations associated with formation of a link in the nomination layer.

**Table 16:** Difference in the number of collocations between nodes to-be-communicators and not-to-be-communicators

Semester	To-be-communicators	Not-to-be-communicators
Semester 1	74.6	25.9
Semester 2	70.4	36.2

#### Step-wise evolution of significant contact formation

After confirming that increasing weight in the communication layer is very likely an intermediate stage preceding the formation of ties in the nomination, we can observe how some edges in the nomination layer emerge gradually over time, from mere collocation to communication finally leading to nomination as cognitively salient contact. In the first semester, we find that students who start communicating in the second semester, but who do not achieve enough cognitive salience to nominate one another in the network survey, have on an average 74.6 collocations, while those who would not communicate have on an average 25.9 collocations. Now out of these communicators in the second semester, the ones who move on to become nominees in the third semester, have on an average 166.2 collocations, while those that do not become nominees have on an average 55.1 collocations in the second semester. So we observe how some of the emergence of nomination links progress from collocation to communication, gradually increasing the number of occasions in which they find themselves in the same place.

#### Are changes in collocation behavior more strongly affected by the dissolution of communication or nomination edges?

In this section, we examine the question of whether collocation behavior is affected more strongly by edge dissolution in the nomination layer or edge decay in the communication layer. To do this, we measure how many nodes with edges continue having collocation after the dissolution of communication, and if this behavior differs from collocation after the dissolution of links in the nomination layer. We observe that 42% of nodes initially connected by a communication link continue to have bluetooth collocations after this edge is dissolved. On the other hand, 93% of dyads continue to experience collocation after a contact is no longer nominated. This implies that communication behavior is more important in determining whether people spend time in the same place than cognitive salience. Conversely, this means that people spend time in the same place with persons that are not cognitively salient to them.

### Analysis of asymmetric nomination edges in relation to the behavioral layers

As established in previous work in social network analysis [19], network links premised on cognitive salience, such as the edges in the NetSense nomination layer, have the property of potentially being *asymmetric*: one person may nominate the other as a cognitively salient contact but the other may fail to reciprocate (*A* nominates *B* but *B* does not nominate *A*). This is contrast to communication edges, which have continuous weights and in which relations can be more or less *reciprocal* but almost never completely asymmetric [20]. Behavioral collocations, by definition, have to be symmetric (if *A* is in the same place as *B*, then *B* is in the same place as *A*) [15].

#### Are there differences in communication and collocation volumes between nodes connected by the asymmetric and symmetric edges in the nomination layer?

We examine if nodes connected by asymmetric and symmetric nomination edges differ in communication and collocation volume. It is reasonable to expect that symmetric edges would have significantly higher levels of communication and collocation [15, 19, 20]. To this end, we examine if nodes connected by asymmetric and symmetric edges in the nomination layer differ in communication and collocation intensities. As shown in Table 17, we observe that, apart from the first semester, there is a significant difference in behaviors of nodes connected by asymmetric and symmetric edges in the nomination layer, with nodes connected by symmetric nominations communicating more frequently than others. In the first semester, the same difference exists, but it is much smaller and visible only if the sum of calls and messages is taken into account. Table 18 shows the differences between the numbers of collocations for nodes connected by the symmetric and asymmetric edges in the nomination layer. As expected, we find that dyads connected by symmetric links in the nomination layer spend more time in the same location than dyads linked by asymmetric nomination links. These results provide strong support for the hypothesis that behavioral links are more intense among dyads connected by mutual nominations than they are for dyads in which one person is more cognitively salient to another than the reverse.

**Table 17:** Difference in communication volumes between nodes connected by asymmetric and symmetric nomination edges

Semester	Asymmetric edges		Symmetric edges	
	No. calls	No. messages	No. calls	No. messages
Semester 1	69	472	58	842
Semester 2	25	638	39	636
Semester 3	40	351	112	2038
Semester 4	10	256	70	1406

**Table 18:** Difference in the numbers of collocations between nodes connected by asymmetric and symmetric nomination edges

Semester	Asymmetric edges	Symmetric edges
Semester 1	108.9	28.6
Semester 2	145.6	37.5

#### Are nodes with asymmetric edges in the nomination layer more likely to be connected by non-reciprocal communication edges?

Next, we examine whether nodes connected by non-reciprocal communication edges are also more likely to have asymmetric communication patterns. We define a non-reciprocal communication edge as one in which one node initiates communications with the other node more often than the reverse [20]. We compare communication imbalance between nodes connected by asymmetric and non-symmetric nomination links. To measure non-reciprocity in the communication layer, we first compute the ratio of the volume of communication in which the source node is the initiator to the volume of communication in which the destination node is the initiator: we call this quantity *One-Sided Communication Factor, OSCF*. We multiply the number of calls by 10, since messages are about 10 times more frequent than calls and add the product to the number of messages. Using this result, we measure the percentage of non-reciprocated communication for nodes connected by both asymmetric and symmetric in the nomination layer.

We find that nodes connected by symmetric nominations have high rates of reciprocal communications. In the first semester only 3% of nodes linked by symmetric nominations have corresponding behavioral edges that count as non-reciprocal according to the criterion defined in “Are nodes with asymmetric edges in the nomination layer more likely to connected by be non-reciprocal communication edges?” section. In comparison, asymmetric edges in the nomination layer are much more likely to be linked corresponding to non-reciprocal edges in the communication layer: In the first semester, the nodes connected by the asymmetric nomination edges were about ten times more likely (31%) to have imbalanced communication than the nodes connected by symmetric nomination edges. Table 19 summarizes these results.

**Table 19:** Difference between symmetric and asymmetric edges in the fractions of having non-reciprocal communication, OSCF

Semester	Asymmetric edges (%)	Symmetric edges (%)
Semester 1	31	3
Semester 2	30	1
Semester 3	39	10
Semester 4	31	6

#### Is behavioral edge dissolution faster for nodes linked by asymmetric edges in the nomination layer?

We examine if nodes connected by asymmetric nomination edges are more likely to experience behavioral edge decay than nodes connected by symmetric nominations. To do so, we measure the survival probabilities of behavioral edges between nodes connected by asymmetric and symmetric edges across all semesters. We find that nodes connected by asymmetric nominations are significantly more likely to experience decay and dissolution in the communication layer than the nodes connected by symmetric edges in the nomination layer in all three semesters.

Nodes joined by asymmetric edges in the nomination layer have dissolution probabilities of 90, 87.5, and 50% in the communication layer in each of the three semesters. In contrast, the corresponding probabilities for dyads joined by symmetric nomination links are 72, 66, and 16% in each of the three semesters. We also observe an overall downward trend in the dissolution probability in the communication layer. Initially, these are very high for the first semester, but they decline steadily over time. However, even in the third semester, nodes connected by asymmetric nomination edges are more than three times more likely to dissolve their behavioral edges than nodes joined by symmetric nominations.

#### Communication behavior profile: the “non-reciprocal sender” profile

We classify nodes that are more likely to be involved in non-reciprocal communication as *non-reciprocal senders*. We then examine the communication behavior profile of these nodes to see if the non-reciprocal sender profile differs from reciprocal sender profile. The goal is to verify if nodes with different communication profiles are more likely to experience their changes in the nomination layer, given the well-known psychological aversion to lack of reciprocity [3, 19, 37]. We find support for the hypothesis that non-reciprocal senders have a larger churn of nominees, in the observation that non-reciprocal senders retain 7, 16, and 38% of their links in the nomination layer, while reciprocal senders retain 25, 50, and 88% of their nomination links in the succeeding semesters.

## Discussion

### Implications of the results

A fundamental question in the analysis of social networks concerns itself with dependencies between links based on cognitive salience, such as those elicited from traditional network surveys, and behavioral linkages indicative of direct communicative exchange, or providing the potential for the formation of close relationships such frequenting the same physical location [3, 12, 37, 38]. Dependencies between cognitive salience, propinquity, and behavioral interactions have been difficult to study in the past, mainly due to lack of availability of dynamic, ecologically valid data in which the temporal dependencies of network ties across these different layers could be examined [9, 15].

In this paper, we have provided a unified empirical treatment of the temporal coevolution of network layers capturing these three key types of human connectivity. Our results provide confirmation for classical lines of network theory [6, 37, 38], while revealing novel insights about the linkage between cognition, communication, and behavior. First, we show that there are systematic dependencies between the cognitive salience of contacts and communication and collocation behavior. All else equal, persons tend to communicate and spend time in the same place as those contacts that are cognitively salient to them. This is particularly the case for those contacts whose cognitive salience persists over time. This is consistent with the idea that cognitive structure is a key determinant of behavioral structure [38], and that frequency of interaction and time-spent together are important components of the concept of tie strength [39]. However, our results also show that just in the same way that cognition is predictive of behavior, behavior is predictive of dynamic changes in the relative salience of contacts. Using state-of-the-art machine-learning techniques we showed that future changes in the cognitive salience of contacts can be predicted from pre-existing (and potential) interactions, both in terms of communication and propinquity behavior.

Previous work on the cognitive salience of social contacts using network surveys has shown that such ties are subject to what has been referred as *decay*; this is the phenomenon whereby a person may nominate another as a cognitively salient contact at time *t* but fail to do so at time $t+1$. While the phenomenon of tie decay is well studied in social network analysis [40], it has not been previously linked to coevolution dynamics of behavioral ties. In this paper we showed that there are systematic links between tie-decay dynamics in terms of cognitive salience with respect to changes in dynamic behavioral layers. For ties that decline in cognitive salience over time, some changes in communication behavior and very minimal changes in collocation behavior occur. This implies that changes in cognitive salience are less predictive of behavioral changes than the reverse. Nevertheless, we do find that cognitive salientties exhibit systematic differences in terms of the *temporal profile* in which behaviors are enacted. Cognitively salient ties tend to be activated (either via communication or collocation) at days of the week or times of the day much more likely to be indicative of informal sociability. One implication of this novel result is that this behavioral temporal signature could be used to predict cognitive salience in the absence of subjective information of social ties.

In examining the linkage between these three different forms of human connectivity, an empirically validated model of the emergence of cognitive salient contacts suggests itself. According to our findings, collocation behavior emerges first, which leads to increases in communication behavior, which then leads to a contact rising up high enough in the cognitive salience hierarchy to be mentioned as a significant tie in a network survey. The process model suggested by our results  is consistent with classic work on the evolution of social contacts from psychology and sociology [19, 37, 41]. We are able to provide systematic empirical evidence for the first time here.

Finally, our empirical work speaks to the fundamental role of asymmetry and non-reciprocity in human connectivity. Previous work has pointed to the fact that the cognitive salience relation can be asymmetric: *A* can be salient to *B* but *B* may not consider *A* salient [3]. In the same way, previous work has shown that communications can be either reciprocal or non-reciprocal [20]. Our empirical work connects these two lines of research for the time, showing that asymmetry at the level of cognitive salience is connected to non-reciprocity in communication behavior in systematic and intuitive ways. All else being equal, asymmetric nominations lead to non-reciprocities in communication. Not only that, asymmetry at the level of cognitive salience predicts tie decay in the communication layer, while also predicting the churning of ties at the level of the individual.

### Limitations and suggestions for future research

The present work advances theory and research in social network analysis, especially with regard to the dynamic coevolution of social ties across multiple connectivity layers. However, our results also open up a variety of questions that cannot be answered given the limitations of the data, and which should be the subject of future work. In particular, moving our framework to a larger population beyond the college student setting, and ascertaining whether our results hold in other human interaction foci (such as work organizations) is important. Linking cognitive salience to other subjective features of social relations, such as emotional closeness, role-relations (e.g., friendship versus kin ties), the exchange of resources (e.g., advice, or emotional support), and looking at how these edge-level variables in the nomination layer interact with communication and collocation behavior also seems like a pertinent subject of future research. Finally, while only suggestive at this stage, expanding the work on asymmetries and non-reciprocities in both cognitive salience and behavioral interactions seems like a promising avenue of future research. This work could look at the individual, dyadic, and contextual correlates of reciprocities and non-reciprocities in interaction as these interact with subjective symmetries and asymmetries in cognitive salience. In all, the work reported here opens up multiple avenues for future work at the intersection of cognition, behavior, and human social networks.
